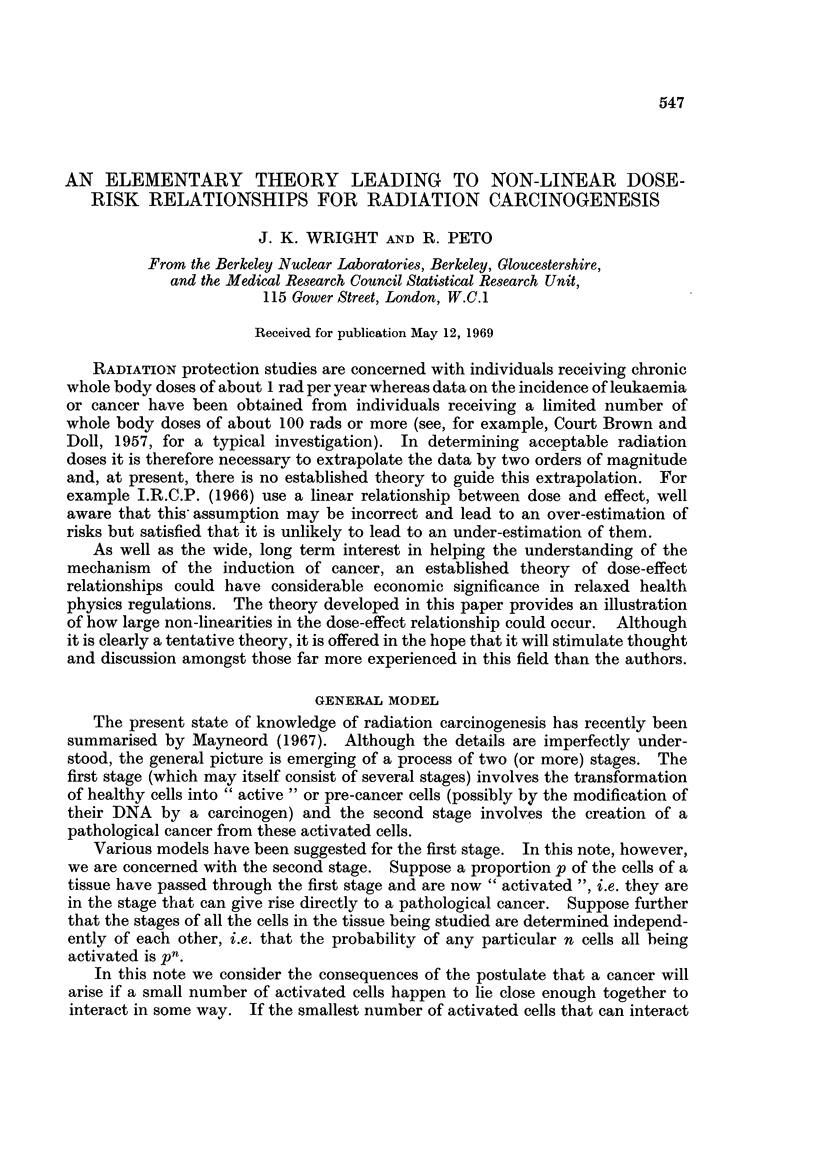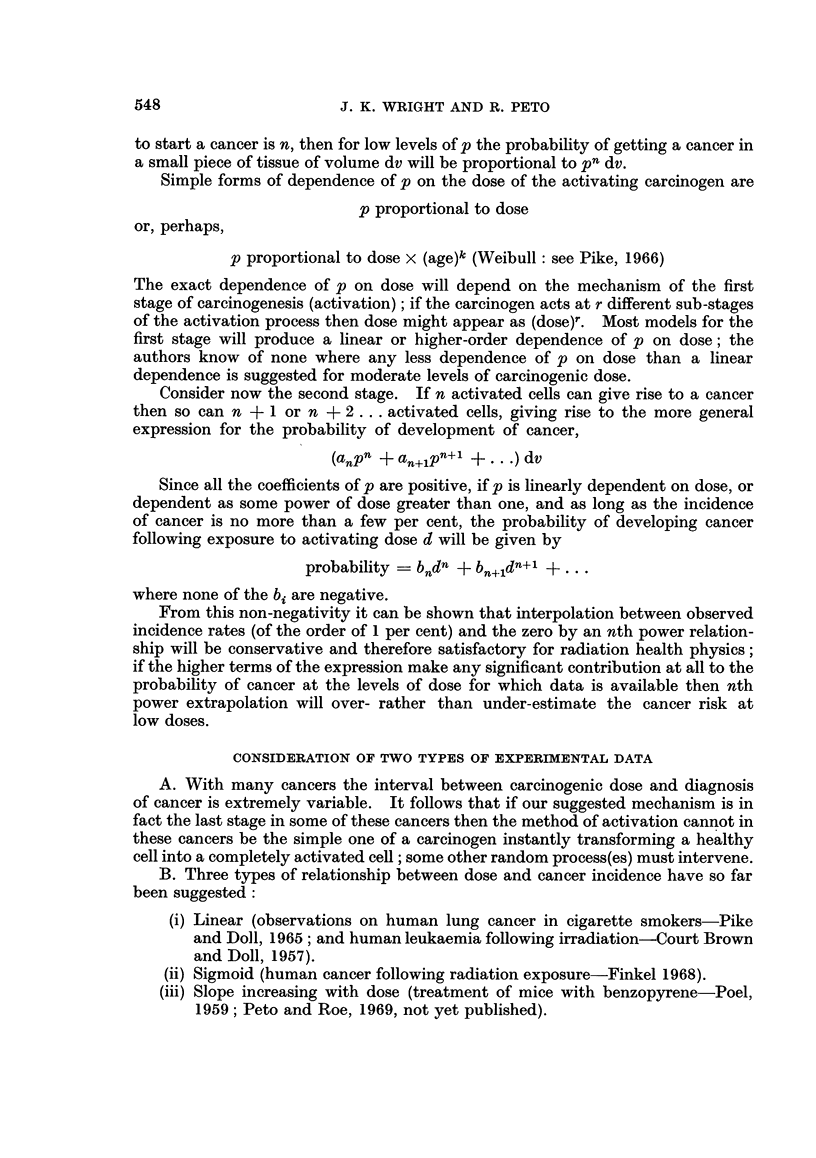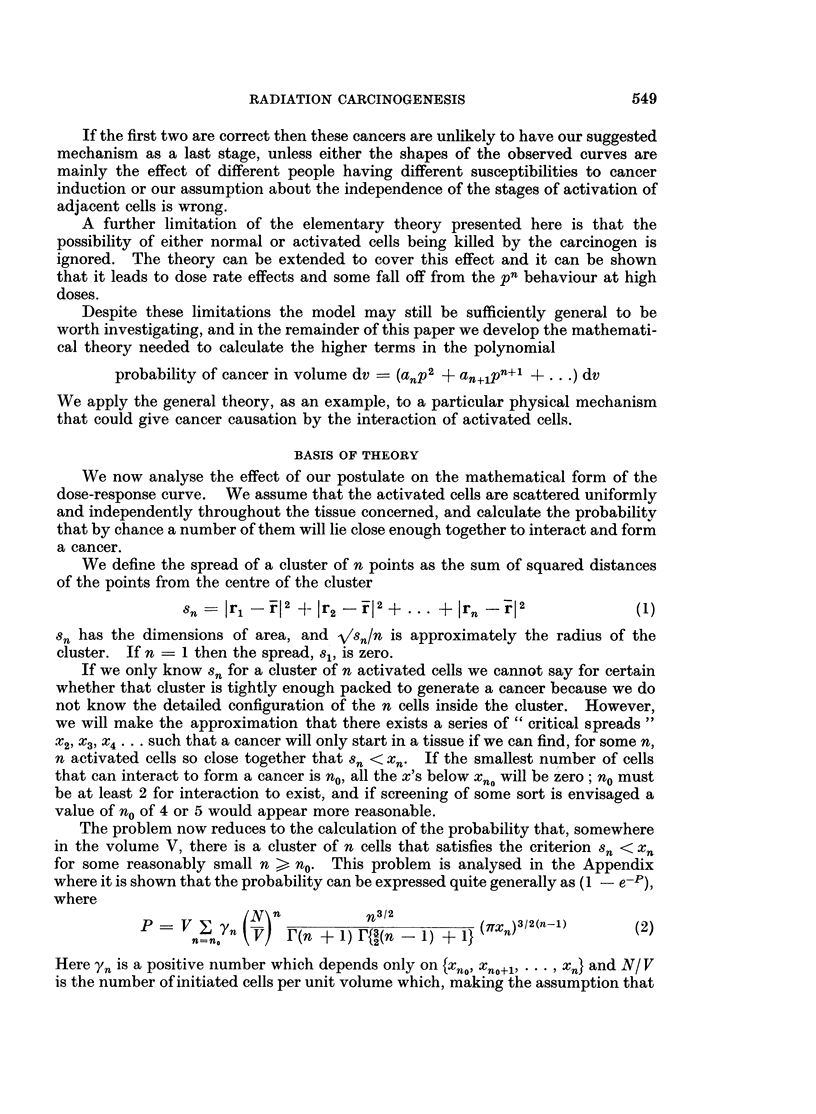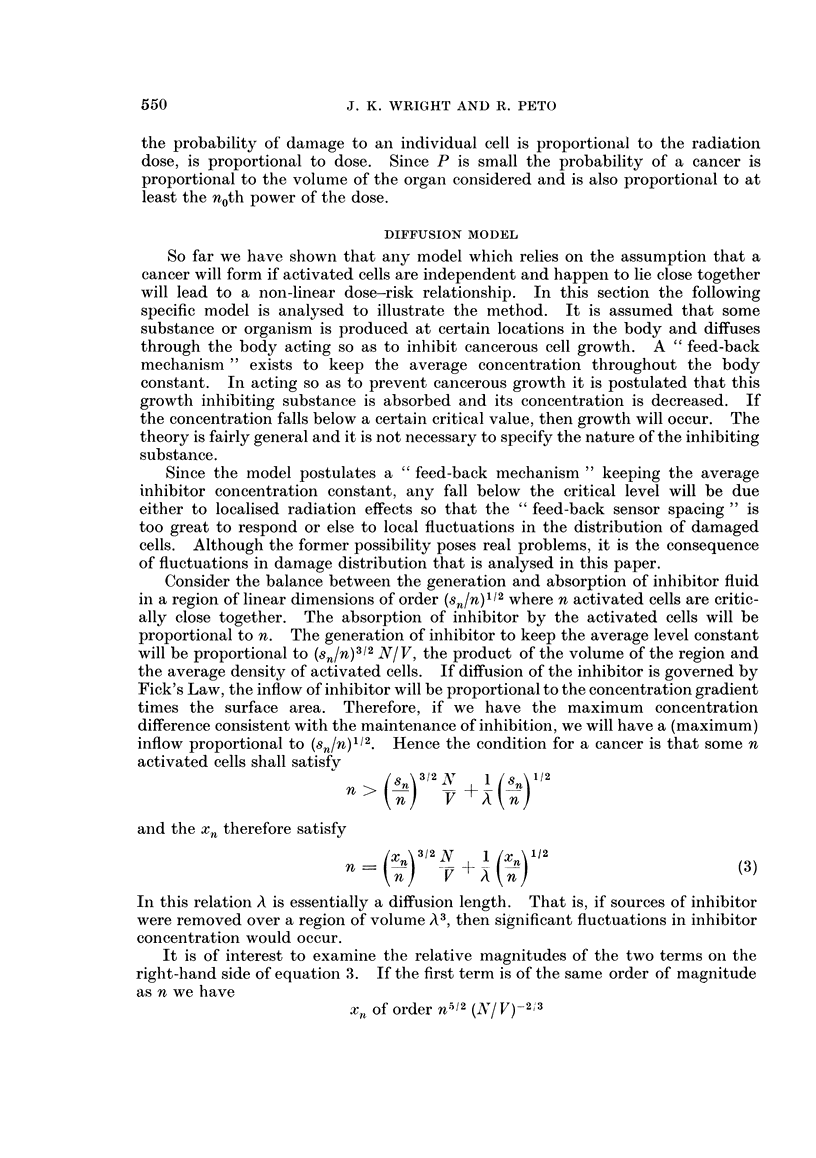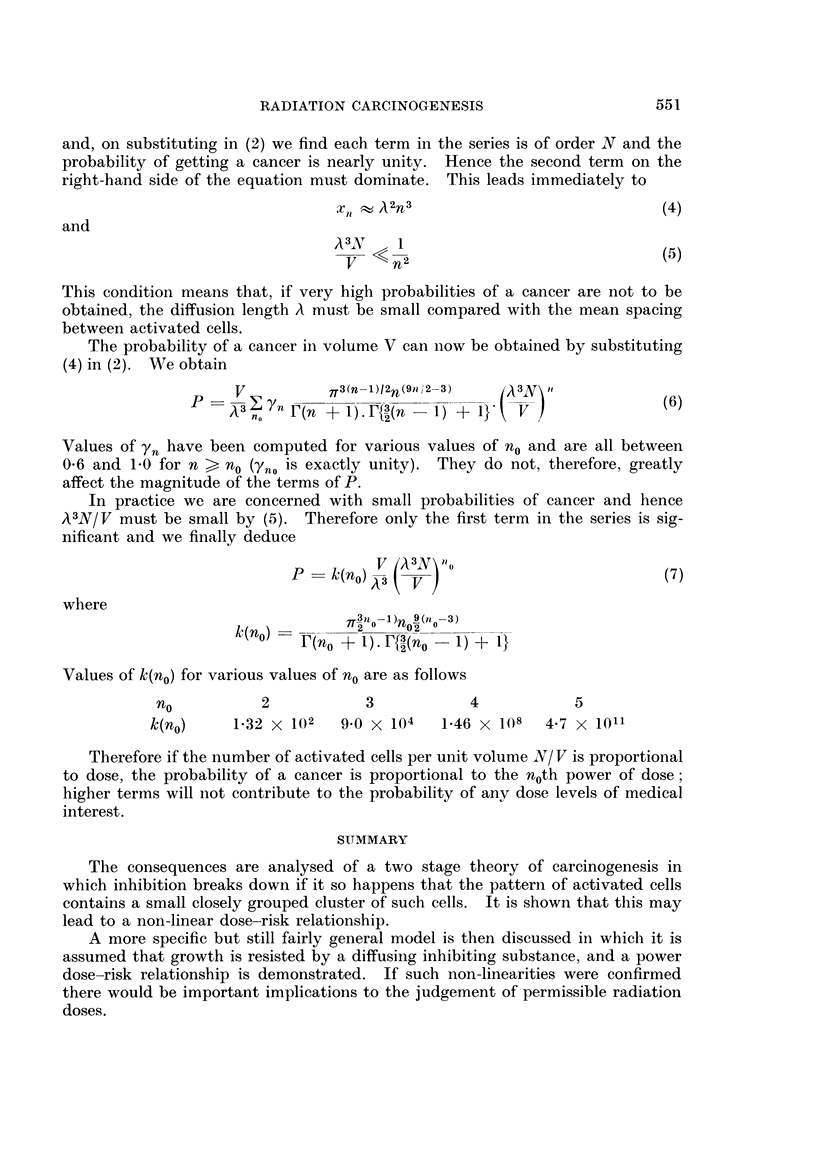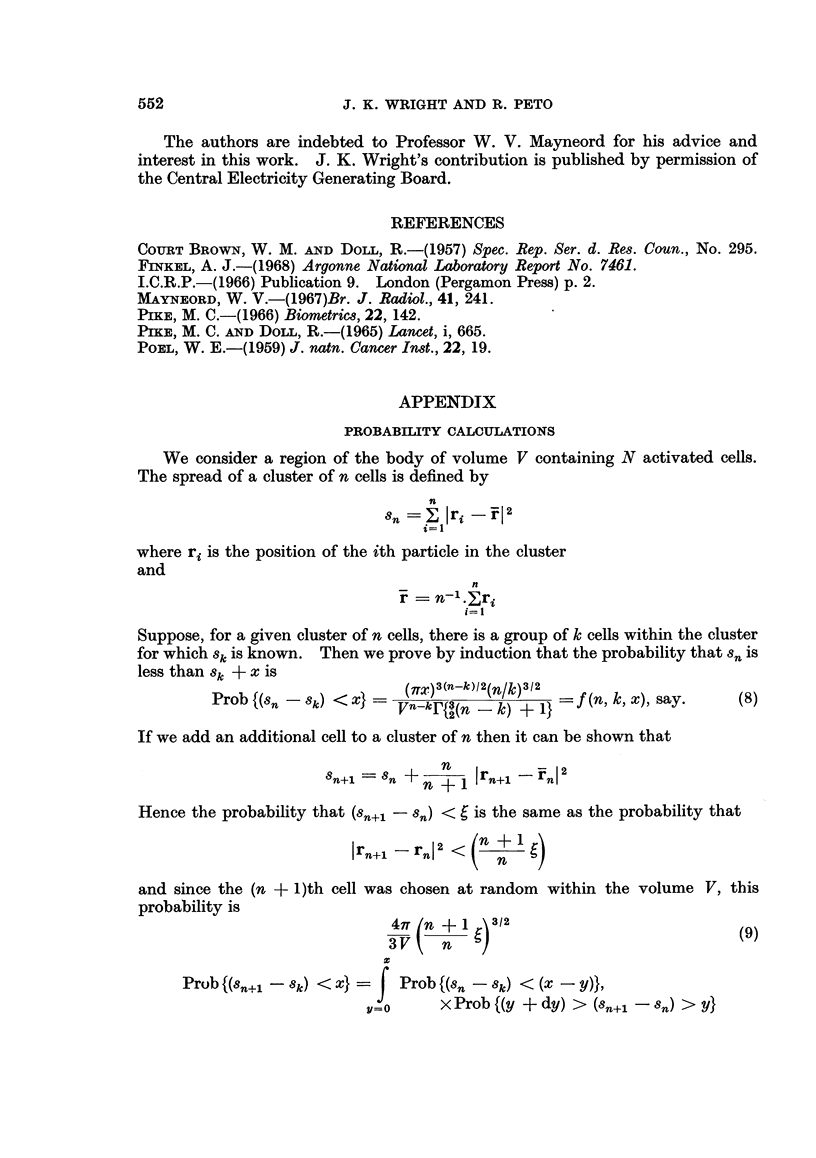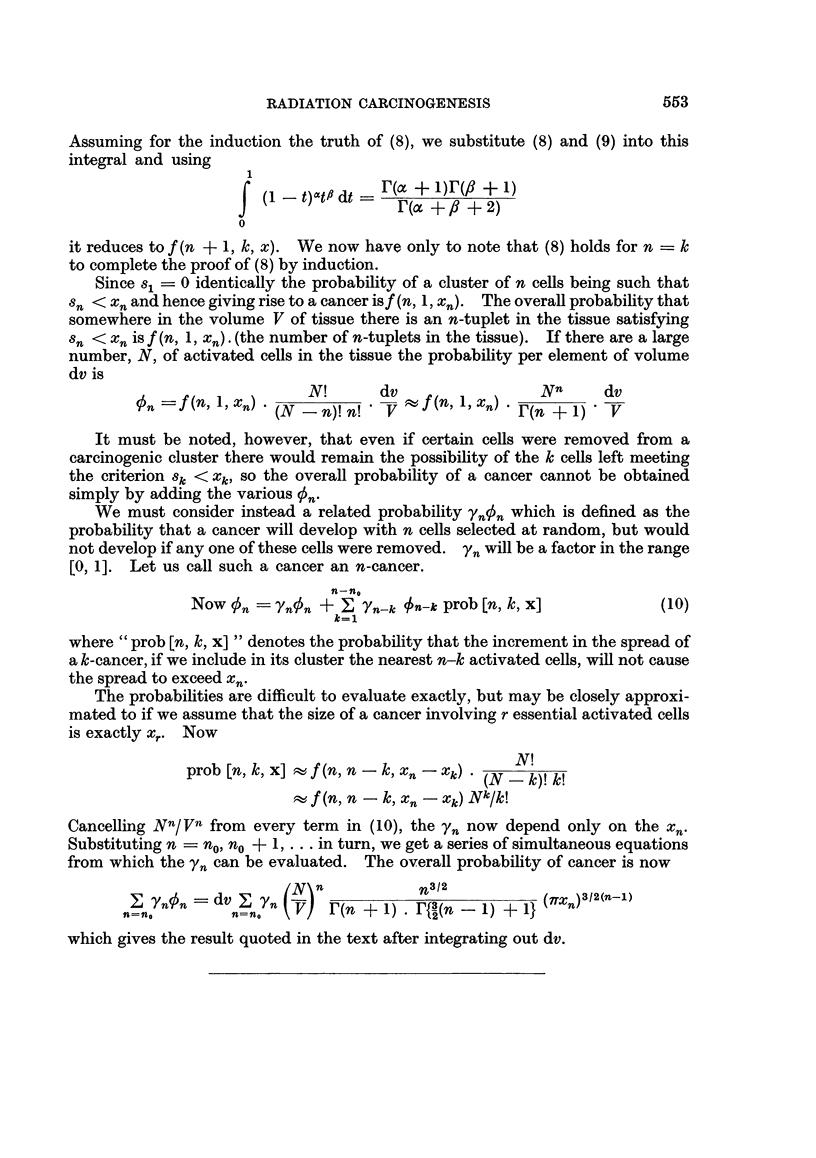# An elementary theory leading to non-linear dose-risk relationships for radiation carcinogenesis.

**DOI:** 10.1038/bjc.1969.67

**Published:** 1969-09

**Authors:** J. K. Wright, R. Peto


### AN ELEMENTARY THEORY LEADING TO NON-LINEAR DOSE-RISK RELATIONSHIPS FOR RADIATION CARCINOGENESIS

J. K. WRIGHT AND R. PETO

*From the Berkeley Nuclear Laboratories, Berkeley, Gloucestershire, and the Medical Research Council Statistical Research Unit, 115 Gower Street, London, W.C.1*

Received for publication May 12, 1969

RADIATION protection studies are concerned with individuals receiving chronic whole body doses of about 1 rad per year whereas data on the incidence of leukaemia or cancer have been obtained from individuals receiving a limited number of whole body doses of about 100 rads or more (see, for example, Court Brown and Doll, 1957, for a typical investigation). In determining acceptable radiation doses it is therefore necessary to extrapolate the data by two orders of magnitude and, at present, there is no established theory to guide this extrapolation. For example I.R.C.P. (1966) use a linear relationship between dose and effect, well aware that this assumption may be incorrect and lead to an over-estimation of risks but satisfied that it is unlikely to lead to an under-estimation of them.

As well as the wide, long term interest in helping the understanding of the mechanism of the induction of cancer, an established theory of dose-effect relationships could have considerable economic significance in relaxed health physics regulations. The theory developed in this paper provides an illustration of how large non-linearities in the dose-effect relationship could occur. Although it is clearly a tentative theory, it is offered in the hope that it will stimulate thought and discussion amongst those far more experienced in this field than the authors.

### GENERAL MODEL

The present state of knowledge of radiation carcinogenesis has recently been summarised by Mayneord (1967). Although the details are imperfectly understood, the general picture is emerging of a process of two (or more) stages. The first stage (which may itself consist of several stages) involves the transformation of healthy cells into " active " or pre-cancer cells (possibly by the modification of their DNA by a carcinogen) and the second stage involves the creation of a pathological cancer from these activated cells.

Various models have been suggested for the first stage. In this note, however, we are concerned with the second stage. Suppose a proportion $p$ of the cells of a tissue have passed through the first stage and are now " activated ", *i.e.* they are in the stage that can give rise directly to a pathological cancer. Suppose further that the stages of all the cells in the tissue being studied are determined independently of each other, *i.e.* that the probability of any particular $n$ cells all being activated is $p^n$.

In this note we consider the consequences of the postulate that a cancer will arise if a small number of activated cells happen to lie close enough together to interact in some way. If the smallest number of activated cells that can interact to start a cancer is $n$, then for low levels of $p$ the probability of getting a cancer in a small piece of tissue of volume d$v$ will be proportional to $p^n$ d$v$.

Simple forms of dependence of $p$ on the dose of the activating carcinogen are

$$p \text{ proportional to dose}$$

or, perhaps,

$$p \text{ proportional to dose} \times (\text{age})^k \text{ (Weibull : see Pike, 1966)}$$

The exact dependence of $p$ on dose will depend on the mechanism of the first stage of carcinogenesis (activation) ; if the carcinogen acts at $r$ different sub-stages of the activation process then dose might appear as (dose)$^r$. Most models for the first stage will produce a linear or higher-order dependence of $p$ on dose ; the authors know of none where any less dependence of $p$ on dose than a linear dependence is suggested for moderate levels of carcinogenic dose.

Consider now the second stage. If $n$ activated cells can give rise to a cancer then so can $n + 1$ or $n + 2$ . . . activated cells, giving rise to the more general expression for the probability of development of cancer,

$$(a_n p^n + a_{n+1} p^{n+1} + \ldots) \, \mathrm{d}v$$

Since all the coefficients of $p$ are positive, if $p$ is linearly dependent on dose, or dependent as some power of dose greater than one, and as long as the incidence of cancer is no more than a few per cent, the probability of developing cancer following exposure to activating dose $d$ will be given by

$$\text{probability} = b_n d^n + b_{n+1} d^{n+1} + \ldots$$

where none of the $b_i$ are negative.

From this non-negativity it can be shown that interpolation between observed incidence rates (of the order of 1 per cent) and the zero by an $n$th power relationship will be conservative and therefore satisfactory for radiation health physics ; if the higher terms of the expression make any significant contribution at all to the probability of cancer at the levels of dose for which data is available then $n$th power extrapolation will over- rather than under-estimate the cancer risk at low doses.

### CONSIDERATION OF TWO TYPES OF EXPERIMENTAL DATA

A. With many cancers the interval between carcinogenic dose and diagnosis of cancer is extremely variable. It follows that if our suggested mechanism is in fact the last stage in some of these cancers then the method of activation cannot in these cancers be the simple one of a carcinogen instantly transforming a healthy cell into a completely activated cell ; some other random process(es) must intervene.

B. Three types of relationship between dose and cancer incidence have so far been suggested :

(i) Linear (observations on human lung cancer in cigarette smokers—Pike and Doll, 1965 ; and human leukaemia following irradiation—Court Brown and Doll, 1957).
(ii) Sigmoid (human cancer following radiation exposure—Finkel 1968).
(iii) Slope increasing with dose (treatment of mice with benzopyrene—Poel, 1959 ; Peto and Roe, 1969, not yet published).

If the first two are correct then these cancers are unlikely to have our suggested mechanism as a last stage, unless either the shapes of the observed curves are mainly the effect of different people having different susceptibilities to cancer induction or our assumption about the independence of the stages of activation of adjacent cells is wrong.

A further limitation of the elementary theory presented here is that the possibility of either normal or activated cells being killed by the carcinogen is ignored. The theory can be extended to cover this effect and it can be shown that it leads to dose rate effects and some fall off from the $p^n$ behaviour at high doses.

Despite these limitations the model may still be sufficiently general to be worth investigating, and in the remainder of this paper we develop the mathematical theory needed to calculate the higher terms in the polynomial

$$\text{probability of cancer in volume } \mathrm{d}v = (a_n p^2 + a_{n+1} p^{n+1} + \ldots)\,\mathrm{d}v$$

We apply the general theory, as an example, to a particular physical mechanism that could give cancer causation by the interaction of activated cells.

### BASIS OF THEORY

We now analyse the effect of our postulate on the mathematical form of the dose-response curve. We assume that the activated cells are scattered uniformly and independently throughout the tissue concerned, and calculate the probability that by chance a number of them will lie close enough together to interact and form a cancer.

We define the spread of a cluster of $n$ points as the sum of squared distances of the points from the centre of the cluster

$$s_n = |\mathbf{r}_1 - \bar{\mathbf{r}}|^2 + |\mathbf{r}_2 - \bar{\mathbf{r}}|^2 + \ldots + |\mathbf{r}_n - \bar{\mathbf{r}}|^2 \tag{1}$$

$s_n$ has the dimensions of area, and $\sqrt{s_n/n}$ is approximately the radius of the cluster. If $n = 1$ then the spread, $s_1$, is zero.

If we only know $s_n$ for a cluster of $n$ activated cells we cannot say for certain whether that cluster is tightly enough packed to generate a cancer because we do not know the detailed configuration of the $n$ cells inside the cluster. However, we will make the approximation that there exists a series of " critical spreads " $x_2, x_3, x_4 \ldots$ such that a cancer will only start in a tissue if we can find, for some $n$, $n$ activated cells so close together that $s_n < x_n$. If the smallest number of cells that can interact to form a cancer is $n_0$, all the $x$'s below $x_{n_0}$ will be zero ; $n_0$ must be at least 2 for interaction to exist, and if screening of some sort is envisaged a value of $n_0$ of 4 or 5 would appear more reasonable.

The problem now reduces to the calculation of the probability that, somewhere in the volume V, there is a cluster of $n$ cells that satisfies the criterion $s_n < x_n$ for some reasonably small $n \geqslant n_0$. This problem is analysed in the Appendix where it is shown that the probability can be expressed quite generally as $(1 - e^{-P})$, where

$$P = V \sum_{n=n_0} \gamma_n \left(\frac{N}{V}\right)^n \frac{n^{3/2}}{\Gamma(n+1)\,\Gamma\{\frac{3}{2}(n-1)+1\}} (\pi x_n)^{3/2(n-1)} \tag{2}$$

Here $\gamma_n$ is a positive number which depends only on $\{x_{n_0}, x_{n_0+1}, \ldots, x_n\}$ and $N/V$ is the number of initiated cells per unit volume which, making the assumption that the probability of damage to an individual cell is proportional to the radiation dose, is proportional to dose. Since $P$ is small the probability of a cancer is proportional to the volume of the organ considered and is also proportional to at least the $n_0$th power of the dose.

### DIFFUSION MODEL

So far we have shown that any model which relies on the assumption that a cancer will form if activated cells are independent and happen to lie close together will lead to a non-linear dose–risk relationship. In this section the following specific model is analysed to illustrate the method. It is assumed that some substance or organism is produced at certain locations in the body and diffuses through the body acting so as to inhibit cancerous cell growth. A " feed-back mechanism " exists to keep the average concentration throughout the body constant. In acting so as to prevent cancerous growth it is postulated that this growth inhibiting substance is absorbed and its concentration is decreased. If the concentration falls below a certain critical value, then growth will occur. The theory is fairly general and it is not necessary to specify the nature of the inhibiting substance.

Since the model postulates a " feed-back mechanism " keeping the average inhibitor concentration constant, any fall below the critical level will be due either to localised radiation effects so that the " feed-back sensor spacing " is too great to respond or else to local fluctuations in the distribution of damaged cells. Although the former possibility poses real problems, it is the consequence of fluctuations in damage distribution that is analysed in this paper.

Consider the balance between the generation and absorption of inhibitor fluid in a region of linear dimensions of order $(s_n/n)^{1/2}$ where $n$ activated cells are critically close together. The absorption of inhibitor by the activated cells will be proportional to $n$. The generation of inhibitor to keep the average level constant will be proportional to $(s_n/n)^{3/2}\,N/V$, the product of the volume of the region and the average density of activated cells. If diffusion of the inhibitor is governed by Fick's Law, the inflow of inhibitor will be proportional to the concentration gradient times the surface area. Therefore, if we have the maximum concentration difference consistent with the maintenance of inhibition, we will have a (maximum) inflow proportional to $(s_n/n)^{1/2}$. Hence the condition for a cancer is that some $n$ activated cells shall satisfy

$$n > \left(\frac{s_n}{n}\right)^{3/2}\frac{N}{V} + \frac{1}{\lambda}\left(\frac{s_n}{n}\right)^{1/2}$$

and the $x_n$ therefore satisfy

$$n = \left(\frac{x_n}{n}\right)^{3/2}\frac{N}{V} + \frac{1}{\lambda}\left(\frac{x_n}{n}\right)^{1/2} \qquad (3)$$

In this relation $\lambda$ is essentially a diffusion length. That is, if sources of inhibitor were removed over a region of volume $\lambda^3$, then significant fluctuations in inhibitor concentration would occur.

It is of interest to examine the relative magnitudes of the two terms on the right-hand side of equation 3. If the first term is of the same order of magnitude as $n$ we have

$$x_n \text{ of order } n^{5/2}\,(N/V)^{-2/3}$$

and, on substituting in (2) we find each term in the series is of order $N$ and the probability of getting a cancer is nearly unity. Hence the second term on the right-hand side of the equation must dominate. This leads immediately to

$$x_n \approx \lambda^2 n^3 \tag{4}$$

and

$$\frac{\lambda^3 N}{V} \ll \frac{1}{n^2} \tag{5}$$

This condition means that, if very high probabilities of a cancer are not to be obtained, the diffusion length $\lambda$ must be small compared with the mean spacing between activated cells.

The probability of a cancer in volume V can now be obtained by substituting (4) in (2). We obtain

$$P = \frac{V}{\lambda^3} \sum_{n_0} \gamma_n \frac{\pi^{3(n-1)/2} n^{(9n/2-3)}}{\Gamma(n+1).\Gamma\{\frac{3}{2}(n-1)+1\}} \cdot \left(\frac{\lambda^3 N}{V}\right)^n \tag{6}$$

Values of $\gamma_n$ have been computed for various values of $n_0$ and are all between 0·6 and 1·0 for $n \geqslant n_0$ ($\gamma_{n_0}$ is exactly unity). They do not, therefore, greatly affect the magnitude of the terms of $P$.

In practice we are concerned with small probabilities of cancer and hence $\lambda^3 N/V$ must be small by (5). Therefore only the first term in the series is significant and we finally deduce

$$P = k(n_0) \frac{V}{\lambda^3} \left(\frac{\lambda^3 N}{V}\right)^{n_0} \tag{7}$$

where

$$k(n_0) = \frac{\pi^{\frac{3}{2}n_0 - 1)} n_0^{\frac{9}{2}(n_0-3)}}{\Gamma(n_0+1).\Gamma\{\frac{3}{2}(n_0-1)+1\}}$$

Values of $k(n_0)$ for various values of $n_0$ are as follows

| $n_0$ | 2 | 3 | 4 | 5 |
|---|---|---|---|---|
| $k(n_0)$ | $1{\cdot}32 \times 10^2$ | $9{\cdot}0 \times 10^4$ | $1{\cdot}46 \times 10^8$ | $4{\cdot}7 \times 10^{11}$ |

Therefore if the number of activated cells per unit volume $N/V$ is proportional to dose, the probability of a cancer is proportional to the $n_0$th power of dose; higher terms will not contribute to the probability of any dose levels of medical interest.

### SUMMARY

The consequences are analysed of a two stage theory of carcinogenesis in which inhibition breaks down if it so happens that the pattern of activated cells contains a small closely grouped cluster of such cells. It is shown that this may lead to a non-linear dose–risk relationship.

A more specific but still fairly general model is then discussed in which it is assumed that growth is resisted by a diffusing inhibiting substance, and a power dose–risk relationship is demonstrated. If such non-linearities were confirmed there would be important implications to the judgement of permissible radiation doses.

The authors are indebted to Professor W. V. Mayneord for his advice and interest in this work. J. K. Wright's contribution is published by permission of the Central Electricity Generating Board.

### REFERENCES

COURT BROWN, W. M. AND DOLL, R.—(1957) *Spec. Rep. Ser. d. Res. Coun.*, No. 295.
FINKEL, A. J.—(1968) *Argonne National Laboratory Report No. 7461.*
I.C.R.P.—(1966) Publication 9. London (Pergamon Press) p. 2.
MAYNEORD, W. V.—(1967)*Br. J. Radiol.*, **41**, 241.
PIKE, M. C.—(1966) *Biometrics*, **22**, 142.
PIKE, M. C. AND DOLL, R.—(1965) *Lancet*, i, 665.
POEL, W. E.—(1959) *J. natn. Cancer Inst.*, **22**, 19.

### APPENDIX

### PROBABILITY CALCULATIONS

We consider a region of the body of volume $V$ containing $N$ activated cells. The spread of a cluster of $n$ cells is defined by

$$s_n = \sum_{i=1}^{n} |\mathbf{r}_i - \bar{\mathbf{r}}|^2$$

where $\mathbf{r}_i$ is the position of the $i$th particle in the cluster and

$$\bar{\mathbf{r}} = n^{-1}.\sum_{i=1}^{n}\mathbf{r}_i$$

Suppose, for a given cluster of $n$ cells, there is a group of $k$ cells within the cluster for which $s_k$ is known. Then we prove by induction that the probability that $s_n$ is less than $s_k + x$ is

$$\text{Prob}\,\{(s_n - s_k) < x\} = \frac{(\pi x)^{3(n-k)/2}(n/k)^{3/2}}{V^{n-k}\Gamma\{\frac{3}{2}(n-k)+1\}} = f(n, k, x), \text{ say.} \quad (8)$$

If we add an additional cell to a cluster of $n$ then it can be shown that

$$s_{n+1} = s_n + \frac{n}{n+1}\,|\mathbf{r}_{n+1} - \bar{\mathbf{r}}_n|^2$$

Hence the probability that $(s_{n+1} - s_n) < \xi$ is the same as the probability that

$$|\mathbf{r}_{n+1} - \mathbf{r}_n|^2 < \left(\frac{n+1}{n}\,\xi\right)$$

and since the $(n + 1)$th cell was chosen at random within the volume $V$, this probability is

$$\frac{4\pi}{3V}\left(\frac{n+1}{n}\,\xi\right)^{3/2} \quad (9)$$

$$\text{Prob}\,\{(s_{n+1} - s_k) < x\} = \int_{y=0}^{x} \text{Prob}\,\{(s_n - s_k) < (x - y)\}, \times \text{Prob}\,\{(y + \mathrm{d}y) > (s_{n+1} - s_n) > y\}$$

Assuming for the induction the truth of (8), we substitute (8) and (9) into this integral and using

$$\int_0^1 (1-t)^{\alpha} t^{\beta}\, dt = \frac{\Gamma(\alpha+1)\Gamma(\beta+1)}{\Gamma(\alpha+\beta+2)}$$

it reduces to $f(n+1, k, x)$. We now have only to note that (8) holds for $n = k$ to complete the proof of (8) by induction.

Since $s_1 = 0$ identically the probability of a cluster of $n$ cells being such that $s_n < x_n$ and hence giving rise to a cancer is $f(n, 1, x_n)$. The overall probability that somewhere in the volume $V$ of tissue there is an $n$-tuplet in the tissue satisfying $s_n < x_n$ is $f(n, 1, x_n)$ . (the number of $n$-tuplets in the tissue). If there are a large number, $N$, of activated cells in the tissue the probability per element of volume $dv$ is

$$\phi_n = f(n, 1, x_n) \cdot \frac{N!}{(N-n)!\, n!} \cdot \frac{dv}{V} \approx f(n, 1, x_n) \cdot \frac{N^n}{\Gamma(n+1)} \cdot \frac{dv}{V}$$

It must be noted, however, that even if certain cells were removed from a carcinogenic cluster there would remain the possibility of the $k$ cells left meeting the criterion $s_k < x_k$, so the overall probability of a cancer cannot be obtained simply by adding the various $\phi_n$.

We must consider instead a related probability $\gamma_n \phi_n$ which is defined as the probability that a cancer will develop with $n$ cells selected at random, but would not develop if any one of these cells were removed. $\gamma_n$ will be a factor in the range [0, 1]. Let us call such a cancer an $n$-cancer.

$$\text{Now } \phi_n = \gamma_n \phi_n + \sum_{k=1}^{n-n_0} \gamma_{n-k}\, \phi_{n-k}\, \text{prob}\,[n, k, \mathbf{x}] \tag{10}$$

where "prob [$n$, $k$, x]" denotes the probability that the increment in the spread of a $k$-cancer, if we include in its cluster the nearest $n$–$k$ activated cells, will not cause the spread to exceed $x_n$.

The probabilities are difficult to evaluate exactly, but may be closely approximated to if we assume that the size of a cancer involving $r$ essential activated cells is exactly $x_r$. Now

$$\begin{aligned}\text{prob}\,[n, k, \mathbf{x}] &\approx f(n, n-k, x_n - x_k) \cdot \frac{N!}{(N-k)!\, k!} \\ &\approx f(n, n-k, x_n - x_k)\, N^k/k!\end{aligned}$$

Cancelling $N^n/V^n$ from every term in (10), the $\gamma_n$ now depend only on the $x_n$. Substituting $n = n_0, n_0 + 1, \ldots$ in turn, we get a series of simultaneous equations from which the $\gamma_n$ can be evaluated. The overall probability of cancer is now

$$\sum_{n=n_0} \gamma_n \phi_n = dv \sum_{n=n_0} \gamma_n \left(\frac{N}{V}\right)^n \frac{n^{3/2}}{\Gamma(n+1)\,.\,\Gamma\{\frac{3}{2}(n-1)+1\}} (\pi x_n)^{3/2(n-1)}$$

which gives the result quoted in the text after integrating out $dv$.